# Osteoporotic Bone Quality Significantly Increases Proximal Stress Concentration: A Comparative Thermoelastic Stress Analysis with Normal Composite Femurs

**DOI:** 10.3390/bioengineering13050496

**Published:** 2026-04-24

**Authors:** Ryunosuke Watanabe, Shota Yasunaga, Fumi Hirose, Koshiro Shimasaki, Tomohiro Yoshizawa, Yasuhiro Homma, Tomofumi Nishino, Hajime Mishima, Yoshihisa Harada

**Affiliations:** 1 Department of Orthopaedic Surgery, Institute of Medicine, University of Tsukuba, Tsukuba 305-8575, Japan; 2 Core Manufacturing Technology Research Institute, National Institute of Advanced Industrial Science and Technology, Tsukuba 305-8565, Japan

**Keywords:** femoral stress distribution, composite femur, osteoporosis, biomechanics, thermoelastic stress analysis

## Abstract

Proximal femoral fractures associated with osteoporosis are an important clinical problem, yet how bone quality independently influences stress distribution remains insufficiently understood. This study aimed to quantitatively compare surface stress distribution between normal and osteoporotic proximal femoral models using thermoelastic stress analysis (TSA). Fourth-generation composite femurs with identical external geometries were subjected to cyclic compressive loading at a 9° adduction angle, with different maximum loads applied to avoid structural failure (normal: 1900 N; osteoporotic: 1000 N). TSA was performed using an infrared lock-in system to obtain surface stress maps, and stress values were evaluated across key proximal regions and along the medial and lateral cortices. The osteoporotic group showed higher maximum stress values in the medial neck (−37.79 vs. −11.52 MPa), lateral neck (24.70 vs. 8.75 MPa), and intertrochanteric crest (−17.98 vs. −6.05 MPa), corresponding to approximately 1.8–3.5-fold increases compared with the normal model values normalized to 1000 N. Mean stress values were also higher by approximately 1.9–2.4-fold across regions. These results suggest that reduced bone quality is associated with increased proximal stress concentration. They may also help guide implant and fixation strategies, including stem selection and fixation configuration, by identifying regions susceptible to stress concentration under different bone quality conditions.

## 1. Introduction

Proximal femoral fractures frequently occur in older adults, and osteoporosis is a major risk factor [1]. Osteoporosis substantially reduces bone strength by decreasing bone density and deteriorating bone microstructure, leading to fractures from external forces such as falls [2,3]. In osteoporotic bone, trabecular thinning, loss of connectivity, and a transition from plate-like to rod-like structures disrupt the three-dimensional trabecular network. These structural changes restrict load transfer pathways and promote local stress concentration, thereby further increasing fracture risk [4]. Therefore, the evaluation of stress distribution is clinically important. However, direct in vivo measurements are limited. In biomechanical research, standardized test specimens are required to perform reproducible experiments and avoid individual differences and ethical constraints. For this purpose, composite bones with uniform external geometries and internal structures are widely used [5,6,7,8]. In particular, the normal bone model (model 3403) and osteoporotic model (model 3503) are designed with different cortical bone thicknesses and cancellous bone densities, thus enabling the replication of the respective bone strength and quality [5]. These composite bones play an important role in clinically oriented research, such as the design of joint prostheses and mechanical evaluation of fixation methods [9,10,11,12,13,14].

Experiments using composite bones have excellent reproducibility and comparability and are useful for evaluating total hip arthroplasty (THA) and fracture treatment methods. However, despite the differences in bone structure and material properties between normal and osteoporotic models, knowledge regarding the resulting differences in stress transfer patterns has not been sufficiently accumulated. Studies have primarily focused on comparing global mechanical parameters such as failure strength and stiffness, with limited qualitative reports on the stress distribution on the bone surface. Clarifying the location and magnitude of stress concentrations is essential for implant design, fixation method evaluation, and fracture analysis. Therefore, a more detailed surface stress analysis is necessary. Conventional methods such as strain gauge measurements are limited to point-specific data and cannot evaluate stress distribution across the entire composite bone. Furthermore, finite element analysis (FEA) is a computer simulation that requires experimental validation. In contrast, this study employs thermoelastic stress analysis (TSA), which enables the evaluation of the full-field surface stress distribution on the composite bone under actual loading conditions.

Evaluations using osteoporotic models simulate age-related changes in bone quality and are directly relevant to fracture risk assessment and treatment selection in older adults. Therefore, clarifying the influence of bone quality differences on stress distribution will contribute to more reliable experimental designs and improve clinical applicability. The appropriate selection of composite bone models and the establishment of stress evaluation methods form a crucial foundation for the interpretation of experimental results.

In this study, we aimed to quantitatively compare the surface stress distribution in normal and osteoporotic composite femurs by using TSA and to clarify the differences in their stress transfer patterns. The findings of this study will provide fundamental knowledge for the appropriate evaluation of the effect of bone quality differences in future biomechanical tests using composite bones, thereby enhancing the validity of model selection and interpretation.

## 2. Materials and Methods

**Figure 1:**
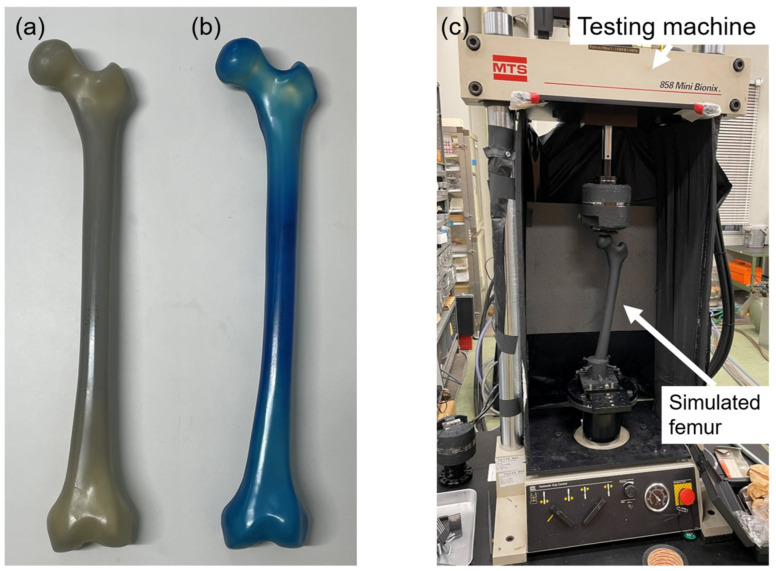
Composite femur models and experimental setup. (**a**) Normal bone model (model 3403) and (**b**) osteoporotic bone model (model 3503). (**c**) Experimental setup for thermoelastic stress analysis.

Fourth-generation composite femurs (Composite Femur^®^; Sawbones, Pacific Research Laboratories, Vashon, WA, USA) were used in this study, specifically model 3403 and model 3503, representing normal and osteoporotic bone conditions, respectively (Figure 1a,b). The structural and mechanical characteristics of these models are summarized in Table 1 based on previous reports [5,15] (Table 1). Cortical thickness of the normal model was measured from CT images at the mid-diaphysis.

The normal model (3403) is designed to replicate the morphology and overall mechanical behavior of a healthy adult femur (height 183 cm, weight 890 N [approx. 90.3 kgf]) [7,8]. It consists of a cortical bone shell made of glass-filled epoxy and cancellous bone made of solid polyurethane foam.

The osteoporotic model has the same external geometry as the normal bone model but features a thinner cortical bone and lower-density polyurethane foam for the cancellous bone. It exhibits mechanical properties that are equivalent to those of osteoporotic cadaveric bones [5].

### 2.1. Thermoelastic Stress Analysis

The thermoelastic effect is a phenomenon in which a material undergoes a slight temperature change under adiabatic conditions because of a change in stress. This effect was first demonstrated by Thomson in 1853 and is expressed as follows [16]:

(1) ΔT = −k·T·Δ(σ_1_ + σ_2_),

where ΔT is the temperature change of the object (K), k is the thermoelastic coefficient (1/Pa), T is the object temperature (K), and Δ(σ_1_ + σ_2_) is the change in the sum of principal stresses (Pa).

The thermoelastic coefficient k is given by the following equation:

(2) k = α/(ρ·Cp),

where α is the coefficient of linear thermal expansion (K^−1^), ρ is the density (kg/m ^3^), and Cp is the specific heat at constant pressure (J/(kg·K)).

TSA is a technique that measures the minute temperature changes (ΔT) occurring when a material is subjected to cyclic loading by using an infrared thermography camera. These temperature changes are then converted into changes in the sum of the principal stresses by using Equation (1). Compressive stress causes a temperature increase, whereas tensile stress causes a temperature decrease. This method cannot separate the individual principal stress components σ_1_ and σ_2_.

TSA has been reported to be an analysis method that correlates well with conventional stress analysis methods, such as the strain gauge method and FEA [17,18,19]. Its application in femoral stress analysis has been advanced by Hyodo et al. [20]. In recent years, TSA has also been widely applied to stress analysis in the orthopedic field [21]. We have applied this method to stress analysis after THA and reported its effectiveness [22,23,24,25].

A linear relationship has been confirmed between the thermoelastic properties of the glass-filled epoxy, used for the cortical bone of the composite femurs in this study, and the change in the sum of the principal stresses and temperature change. A temperature change of 1 K corresponds to a change of approximately 227 MPa in the sum of the principal stresses [16].

### 2.2. Specimen Preparation and TSA

Six normal and six osteoporotic composite femurs were used in this study. The distal femur was resected 6 cm proximal to the femoral condyles and rigidly fixed to a specimen holder using bolts and cement, establishing a fixed boundary condition at the distal end. The femur was then aligned in 9° of varus to simulate the physiological adduction angle during single-leg stance [26]. To ensure uniform emissivity, the entire bone surface was evenly coated with a matte-black, heat-resistant paint (ASAHIPEN Corporation, Osaka, Japan).

The specimen holder containing the femur was rigidly fixed to the base of a hydraulic servo-testing machine (MiniBionix 858; MTS Systems Corporation, Eden Prairie, MN, USA), and a sinusoidal compressive load (5 Hz) was applied vertically to the femoral head. A load of 100–1900 N was applied to the normal bone models. This setting corresponds to a single-leg stance during level walking, with the maximum load being approximately twice the standard weight of 890 N for the composite bone [27]. By contrast, a load of 100–1000 N was applied to the osteoporotic models. This range was based on the loading settings used for cadaveric bones in previous studies, with a maximum load of approximately twice the value [5]. The loading ranges differed between the two models due to differences in structural strength and the need to avoid specimen failure. Because different maximum loads were used, direct quantitative comparison of absolute stress magnitudes between the two models should be interpreted with caution.

Before measurement, each specimen was subjected to over 200 pre-loading cycles to ensure that the load waveform and temperature changes had stabilized. Subsequently, 4000 infrared images corresponding to 100 cycles were captured using an infrared stress measurement system (CPA-SC7500; FLIR Systems Inc., Wilsonville, OR, USA) (Figure 1c). The shooting distance was approximately 1.0 m, and measurements were conducted in a constant-temperature, light-shielded environment to avoid the influence of ambient airflow and external light.

The captured images were processed using lock-in analysis synchronized with the load amplitude by using infrared stress measurement software (TSAvis; Ken Automation Inc., Yokohama, Japan). On the basis of the image data obtained from four directions (anterior, posterior, medial, and lateral), the thermoelastic stress distribution corresponding to the sum of principal stresses on the bone surface was calculated.

### 2.3. Outcomes

**Figure 2:**
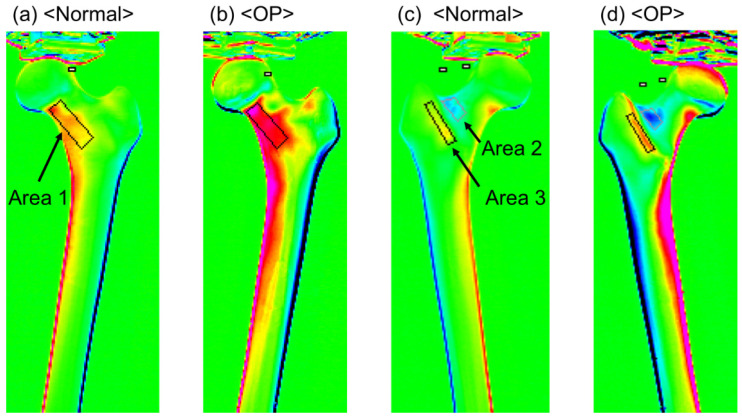
Thermoelastic stress distribution maps of the simulated femur. (**a**,**b**) Stress distribution on the anterior surface showing the medial femoral neck region (Area 1) in the normal and osteoporotic models. (**c**,**d**) Stress distribution on the posterior surface showing the lateral femoral neck region (Area 2) and the intertrochanteric ridge region (Area 3) in the normal and osteoporotic models.

The stress characteristics of each region were evaluated using the stress distribution images of the composite femur surfaces obtained using TSA. First, regions with confirmed stress concentrations were identified, and three areas were selected for analysis: the medial neck from the anterior view (Area 1), the lateral neck from the posterior view (Area 2), and the intertrochanteric crest from the posterior view (Area 3) (Figure 2). For each area, a defined region was set, and the stress values corresponding to the sum of the principal stresses were calculated for Areas 1 (293 pixels), 2 (97 pixels), and 3 (137 pixels). The maximum and mean values were compared between the normal bone model (normal group) and the osteoporotic model (OP group). Furthermore, stress curves were created along the bone axis from the center of the lesser trochanter to the distal diaphysis on the basis of the medial and lateral stress distribution images, and the maximum stress values on these curves were extracted. These indicators were also compared between the two groups to evaluate the effect of bone quality differences on stress transfer characteristics. In addition, the mean stress values and stress curves of the normal model were normalized to a load of 1000 N, and these normalized data were also used for comparison with the OP model.

### 2.4. Statistical Analysis

The Mann–Whitney U test was used to verify the difference in the maximum stress values between the two groups. A linear mixed model (LMM) was used to evaluate group differences in stress values across the three regions (Areas 1–3). The normal and OP groups were set as a fixed effect, and the variability among individual specimens was included as a random effect. Statistical comparisons were performed using the original (non-normalized) data obtained under a maximum load of 1900 N for the normal group and 1000 N for the OP group. The significance level was set at *p* < 0.05.

All statistical analyses were performed using SPSS Statistics version 30.0 (IBM Corp., Armonk, NY, USA).

## 3. Results

**Figure 3:**
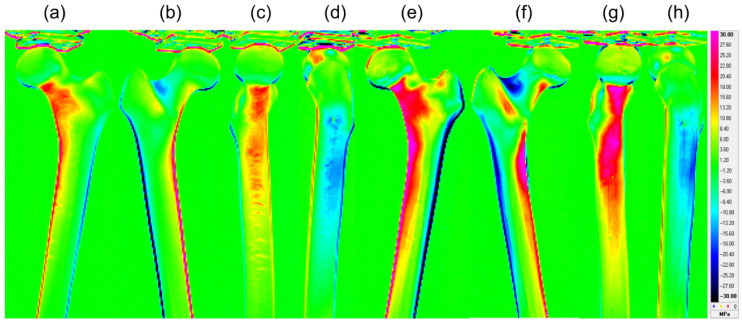
Representative thermoelastic stress distribution figures of the normal and osteoporotic simulated femora. Figures (**a**–**d**) display the anterior, posterior, medial, and lateral surfaces of the normal model, whereas figures (**e**–**h**) show the corresponding surfaces of the osteoporotic model. The red and blue regions indicate high compressive stress and high tensile stress, respectively.

The stress values obtained in this study indicate tensile stress for positive values and compressive stress for negative values, with larger absolute values representing greater stress on the respective parts. Figure 3 shows the representative stress distribution maps (anterior, posterior, medial, and lateral) for normal and osteoporotic bone models. The OP group had higher absolute stress values, primarily around the neck and intertrochanteric regions, and wider high-stress areas than the normal group.

In the comparison of maximum stress values, the OP group showed significantly higher values than the normal group in all evaluated regions: the medial neck (Area 1), lateral neck (Area 2), intertrochanteric crest (Area 3), medial diaphysis, and lateral diaphysis (*p* = 0.002 for all). The analysis using LMM showed that the mean stress in the OP group was significantly higher than that in the normal group across Areas 1–3 (*p* < 0.05) (Table 2).

**Figure 4:**
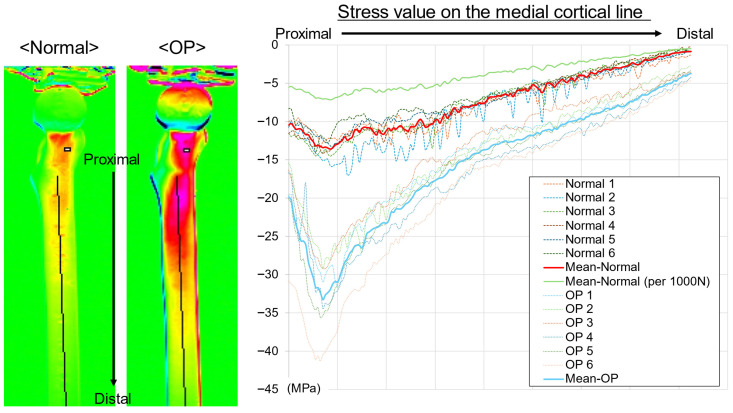
Thermoelastic stress distribution along the medial cortical line. Stress values were extracted from the mid–lesser trochanter to the distal diaphysis in the normal and OP models. Negative values indicate compressive stress. The OP model demonstrated greater compressive stress concentration, particularly distal to the lesser trochanter. Thin lines represent individual specimens, bold lines indicate group mean curves, and the green line shows the mean normal curve normalized to 1000 N. Stress values were calculated using a calibration factor of 227 MPa/K.

On the medial aspect, stress curves were plotted along the center of the bone axis from the lesser trochanter to the distal diaphysis (Figure 4). In both groups, the greatest compressive stress (lowest stress value) occurred just below the lesser trochanter, and the compressive stress gradually decreased distally. A clear difference was observed between the groups at the maximum compression point below the lesser trochanter, with the OP group showing greater compressive stress (lower stress value) than the normal group. Near the mid-diaphysis, the OP group showed greater compressive stress than the normal group; however, the difference was smaller than that in the proximal region. Although the individual curves showed variability, the group mean curves were clearly separated, with the OP group consistently exhibiting greater compressive stress than the normal group. After normalization to 1000 N, the compressive stress values in the normal group were lower, resulting in a greater apparent difference between the groups.

**Figure 5:**
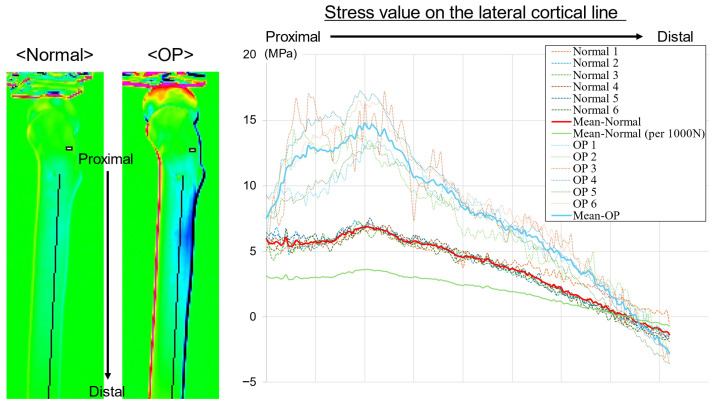
Thermoelastic stress distribution along the lateral cortical line. Stress values were extracted from the mid–lesser trochanter to the distal diaphysis in the normal and OP models. Positive values indicate tensile stress, and negative values indicate compressive stress. The OP model demonstrated greater tensile stress concentration, particularly distal to the lesser trochanter. Thin lines represent individual specimens, bold lines indicate group mean curves, and the green line shows the mean normal curve normalized to 1000 N. Stress values were calculated using a calibration factor of 227 MPa/K.

In the lateral aspect, the stress curves were plotted along the bone axis from the center of the lesser trochanter to the distal diaphysis (Figure 5). In both groups, the highest tensile stress was observed near the subtrochanteric region, after which the stress value gradually decreased distally. A difference was observed between the groups at the peak in the subtrochanteric region, with the OP group exhibiting a higher stress value (greater tensile stress) than the normal group. Near the mid-diaphysis, the stress value in the OP group was also higher than that in the normal group; however, the difference between the groups was smaller than that in the proximal region. Although the individual curves varied, the group mean curves were separated over the entire section, with the OP group consistently exhibiting higher stress values than the normal group. After normalization to 1000 N, the tensile stress values in the normal group were lower, with a greater apparent difference between the groups.

## 4. Discussion

This study demonstrates that differences in bone quality are associated with differences in stress distribution across the proximal femur. In our biomechanical testing using TSA, the osteoporotic composite femur model exhibited significantly higher maximum and mean stress values than the normal model in all key regions, including the femoral neck, intertrochanteric crest, and diaphysis. Stress curves along the medial and lateral cortices showed separation between the groups across the measured length, with the OP group exhibiting greater medial compression and lateral tension than the normal group. Differences were observed in the proximal femur, particularly around the lesser trochanter, a region important for load bearing.

The higher stress values in the OP group may be related to its inherent structural characteristics. This finding was observed despite the OP group being subjected to a lower maximum load than the normal group (1000 N vs. 1900 N). The observation that higher stresses were generated under a lower applied load suggests that reduced bone quality may be associated with increased local stress concentration. The thinner cortex and lower-density cancellous bone of the model likely contributed to a weaker composite structure. This reduced structural integrity may limit the ability of the bone to distribute applied loads efficiently, which may contribute to stress concentration. The increased compressive stress on the medial calcar region may reflect a greater load burden on the medial column, whereas the increased tensile stress on the lateral aspect may indicate that reduced cortical support enhances the effects of the bending moment. This pattern of load transfer may explain the observed trends.

The finding that the OP group consistently showed higher stress values, mainly in the proximal region, aligns with the characteristics of increased stress in osteoporotic bones reported in previous studies. FEA has shown that the peak stress in osteoporotic femurs is 33–45% higher than that in healthy bones [28], with high-stress areas expanding, particularly in the neck and intertrochanteric regions [29]. In the present study, higher stress values were observed in the OP group across the medial and lateral neck and intertrochanteric regions, showing a distribution pattern similar to that reported previously. Peak stress values were approximately 1.8–3.5 times higher than those in the normalized model, and mean stress values were approximately 1.9–2.4 times higher across regions. Compared with previous finite element studies, the magnitude of the difference appeared greater, which may reflect differences in modeling approaches, including material representation, structural characteristics, and loading conditions. These findings support the association between reduced bone quality and increased stress concentration, particularly in the proximal femur.

The characteristics of osteoporotic bone, including reduced cancellous bone mass and a shift in load towards the cortical bone, were also reflected in the stress distribution of the model bones used in this study [4]. High-resolution FEA has shown that the strain in osteoporotic femurs is, on average, approximately 70% higher and more heterogeneous than that in healthy bones [3]. This is because the disruption of the cancellous structure limits the load paths, thus leading to local stress concentrations. Furthermore, load analysis during falls revealed that osteoporotic femurs exhibit higher von Mises stress in proximal regions, such as the neck and trochanteric areas [2,30]. In the current study, despite using composite bones with identical geometries and applying a lower load, the OP group showed higher compressive and tensile stresses in similar regions, with differences between the groups observed in the proximal femur. These results demonstrate that the stress concentration characteristics observed in real bones can be reproduced in an experimental setting by using model bones, thus supporting the idea that composite bones can appropriately reflect the changes in load transfer associated with differences in bone quality.

This study enables independent evaluation of the effect of bone quality differences on femoral surface stress distribution by using standardized composite bones with identical external geometry but differing bone quality. In conventional studies using FEA or cadaveric bones, multiple factors, such as shape and bone density, change simultaneously, thus making it difficult to clearly isolate the influence of bone quality itself. In the current study, analysis of the effect of reduced bone quality on stress transfer patterns was possible by keeping the bone structure and geometric conditions constant and comparing the normal and OP groups.

Another strength of this study is the use of TSA to visualize the surface stress over the entire circumference of the femur and derive stress curves along the medial and lateral cortices. This allowed continuous demonstration of detailed stress changes, such as high stress concentration in the proximal part, stress attenuation towards the distal part, and the persistence of stress differences between the medial and lateral sides. These features are consistent with the stress distribution trends reported in studies on real bones and provide new insights that demonstrate the ability of composite bone tests to appropriately reflect differences in bone quality. Although TSA measures only surface stresses, the observed patterns may provide indirect insight into load transfer, as they are influenced by internal structural behavior. However, this relationship is not direct and should be interpreted together with volumetric approaches such as FEA.

This study has several limitations. First, composite femurs have a simplified structure and do not fully replicate the complex architecture and material heterogeneity of living bone. Second, a single femoral geometry representing an adult male was used. Therefore, the findings should be interpreted as a mechanistic comparison under controlled conditions and are not directly predictive of fracture risk across different sexes, ages, or anatomical morphologies. Anatomical variability may influence stress distribution patterns. Third, different maximum loads were applied to avoid structural failure, which may act as a confounding factor and limit direct quantitative comparison between groups. Fourth, the loading condition was simplified to simulate single-leg stance and does not represent more complex physiological loading. Fifth, TSA measures surface stress and does not provide information on internal stress states; although surface patterns may reflect underlying load transfer, they cannot fully represent three-dimensional stress distribution as in FEA. Finally, the sample size was relatively small. Despite these limitations, this study provides fundamental insights into the biomechanical effects of bone quality under controlled conditions.

## 5. Conclusions

We compared the effects of bone quality differences on surface stress distribution using composite femurs with identical external geometries through TSA. The OP group showed higher compressive and tensile stresses than the normal group, mainly in the proximal region, with differences observed in stress curves along the bone axis. These trends are generally consistent with stress concentration and load transfer patterns reported in osteoporotic femurs, suggesting that the composite models may reflect aspects of bone quality-related stress responses. These findings may help guide implant and fixation strategies, including stem selection and fixation configuration, by identifying regions susceptible to stress concentration under different bone quality conditions.

## Figures and Tables

**Table 1:** Comparison of composite femur 3403 and 3503.

	Normal Model (3403)	Osteoporotic Model (3503)
Cortical Thickness (Mid-Diaphysis)	5.1–8.9 mm (Average: 6.3 mm)	3.1–4.9 mm (Average: 4.5 mm)
Elastic Modulus (Cortical)	16.0 GPa	5.2 GPa
Cancellous Bone Density	0.27 g/cm^3^ (17 pcf)	0.16 g/cm^3^ (10 pcf)

**Table 2:** Comparison of the peak and mean thermoelastic stress values between the normal and OP groups.

	Normal Group (*n* = 6)	Normal (Per 1000 N)	OP Group (*n* = 6)	*p* Value
Peak value (Median [IQR])				
Medial femoral neck (Area 1)	−21.89 [2.94]	−11.52 [1.54]	−37.79 [9.32]	0.002 *
Lateral femoral neck (Area 2)	16.62 [2.71]	8.75 [1.42]	24.70 [5.10]	0.002 *
Intertrochanteric crest (Area 3)	−11.49 [2.27]	−6.05 [1.19]	−17.98 [2.47]	0.002 *
Medial diaphysis	−14.10 [1.77]	−7.42 [0.93]	−33.01 [7.74]	0.002 *
Lateral diaphysis	7.22 [0.50]	3.80 [0.26]	15.61 [3.81]	0.002 *
Mean value (Mean ± SD)				
Medial femoral neck (Area 1)	−13.66 ± 2.67	−7.19 ± 1.40	−24.16 ± 4.74	<0.001 **
Lateral femoral neck (Area 2)	12.13 ± 2.73	6.38 ± 1.43	18.80 ± 3.65	<0.001 **
Intertrochanteric crest (Area 3)	−7.34 ± 1.96	−3.86 ± 1.03	−12.90 ± 2.33	<0.001 **

Data are presented as median [interquartile range] and mean ± standard deviation. Positive and negative values represent tensile and compressive stresses, respectively. normal (per 1000 N) values were calculated by dividing the 1900 N data by 1.9 for both median and mean metrics to allow direct comparison with the OP group. *p* values indicate significant differences between the normal (1900 N) and OP (1000 N) experimental groups. Statistical tests: * Mann–Whitney U test; ** linear mixed model. IQR: interquartile range; SD: standard deviation.

## Data Availability

The data that support the findings of this study are available from the corresponding author upon reasonable request.

## Abbreviations

The following abbreviations are used in this manuscript:

THA	Total Hip Arthroplasty
FEA	Finite Element Analysis
TSA	Thermoelastic Stress Analysis
LMM	Linear Mixed Model

## References

[1] Cummings S.R., Melton L.J. (2002). Epidemiology and outcomes of osteoporotic fractures. Lancet.

[2] Verhulp E., van Rietbergen B., Huiskes R. (2008). Load distribution in the healthy and osteoporotic human proximal femur during a fall to the side. Bone.

[3] Van Rietbergen B., Huiskes R., Eckstein F., Rüegsegger P. (2003). Trabecular bone tissue strains in the healthy and osteoporotic human femur. J. Bone Miner. Res..

[4] Wang H., Zhang Y., Ren C., Ding K., Zhang Q., Zhu Y., Chen W. (2023). Biomechanical properties and clinical significance of cancellous bone in proximal femur: A review. Injury.

[5] Gluek C., Zdero R., Quenneville C.E. (2020). Evaluating the mechanical response of novel synthetic femurs for representing osteoporotic bone. J. Biomech..

[6] Elfar J.J., Menorca R.M., Reed J.D., Stanbury S. (2014). Composite bone models in orthopaedic surgery research and education. J. Am. Acad. Orthop. Surg..

[7] Heiner A.D. (2008). Structural properties of fourth-generation composite femurs and tibias. J. Biomech..

[8] Heiner A.D., Brown T.D. (2001). Structural properties of a new design of composite replicate femurs and tibias. J. Biomech..

[9] Morishima T., Ginsel B.L., Choy G.G.H., Wilson L.J., Whitehouse S.L., Crawford R.W. (2014). Periprosthetic fracture torque for short versus standard cemented hip stems: An experimental in vitro study. J. Arthroplast..

[10] Windell L., Kulkarni A., Alabort E., Barba D., Reed R., Singh H.P. (2021). Biomechanical comparison of periprosthetic femoral fracture risk in three femoral components in a Sawbone model. J. Arthroplast..

[11] Watanabe R., Nishino T., Yoshizawa T., Hirose F., Yasunaga S., Shimasaki K., Mishima H. (2025). Biomechanical comparison of cemented versus cementless fixation in intraoperative femoral fractures during total hip arthroplasty. Arch. Orthop. Trauma Surg..

[12] Takegami Y., Seki T., Osawa Y., Imagama S. (2022). Comparison of periprosthetic femoral fracture torque and strain pattern of three types of femoral components in experimental model. Bone Jt. Res..

[13] Hashimoto K., Nakamura Y., Takahashi N., Morishima T. (2025). Comparison of fracture resistance using two different major cemented stems in osteoporotic bone models. J. Clin. Med..

[14] Restrepo D.J., Guarin Perez S.F., Tai T.W., Zhao G., Tsukamoto I., Labott J.R., Hooke A.W., Zhao C., Sierra R.J. (2026). Biomechanical evaluation of cemented composite beam and cementless collared and collarless triple taper femoral stems with and without prophylactic calcar wiring. J. Arthroplast..

[15] Zdero R., Brzozowski P., Schemitsch E.H. (2023). Biomechanical properties of artificial bones made by Sawbones: A review. Med. Eng. Phys..

[16] Thomson W. (1853). On the dynamical theory of heat, with numerical results deduced from Mr Joule’s equivalent of a thermal unit, and M. Regnault’s observations on steam. Trans. R. Soc. Edinb..

[17] Bougherara H., Rahim E., Shah S., Dubov A., Schemitsch E.H., Zdero R. (2011). A preliminary biomechanical assessment of a polymer composite hip implant using an infrared thermography technique validated by strain gage measurements. J. Biomech. Eng..

[18] Shah S., Bougherara H., Schemitsch E.H., Zdero R. (2012). Biomechanical stress maps of an artificial femur obtained using a new infrared thermography technique validated by strain gages. Med. Eng. Phys..

[19] Bougherara H., Saleem M., Shah S., Toubal L., Sarwar A., Schemitsch E.H., Zdero R. (2018). Stress analysis of a carbon fiber-reinforced epoxy plate with a hole undergoing tension: A comparison of finite element analysis, strain gages, and infrared thermography. J. Compos. Mater..

[20] Hyodo K., Inomoto M., Ma W., Miyakawa S., Tateishi T. (2001). Thermoelastic femoral stress imaging for experimental evaluation of hip prosthesis design. JSME Int. J. C Mech. Syst. Mach. Elem. Manuf..

[21] Zdero R., Brzozowski P., Schemitsch E.H. (2023). Biomechanical stress analysis using thermography: A review. J. Biomech..

[22] Takehashi H., Nishino T., Mishima H., Wada H., Yamazaki M., Hyodo K. (2021). Stress distribution of cementless stems with unique flanges in a rectangular cross-section: Thermoelastic stress imaging study. J. Rural. Med..

[23] Watanabe R., Mishima H., Takehashi H., Wada H., Totsuka S., Nishino T., Yamazaki M., Hyodo K. (2022). Stress analysis of total hip arthroplasty with a fully hydroxyapatite-coated stem: Comparing thermoelastic stress analysis and CT-based finite element analysis. Acta Bioeng. Biomech..

[24] Watanabe R., Mishima H., Takehashi H., Wada H., Totsuka S., Nishino T., Yamazaki M. (2024). Bone mineral density changes around the stem correlate with stress changes after total hip arthroplasty: A study using thermoelastic stress analysis. J. Exp. Orthop..

[25] Shimasaki K., Watanabe R., Nishino T., Yoshizawa T., Hirose F., Yasunaga S., Mishima H., Harada Y. (2025). Optimising stem selection for conversion total hip arthroplasty following femoral trochanteric fracture surgery: An exploratory study using thermoelastic stress analysis. HIP Int..

[26] Kapandji I.A. (2019). The Physiology of the Joints.

[27] Bergmann G., Deuretzbacher G., Heller M., Graichen F., Rohlmann A., Strauss J., Duda G. (2001). Hip contact forces and gait patterns from routine activities. J. Biomech..

[28] Lotz J.C., Cheal E.J., Hayes W.C. (1995). Stress distributions within the proximal femur during gait and falls: Implications for osteoporotic fracture. Osteoporos. Int..

[29] Mirzaei M., Jafari R., Allaveisi F., Jafari H., Alavi F. (2023). Fracture analysis of healthy and osteoporotic femora using clinical CT images, phantomless densitometry, and linear FE method. J. Orthop. Res..

[30] Yano S., Matsuura Y., Hagiwara S., Nakamura J., Kawarai Y., Suzuki T., Kanno K., Shoda J., Tsurumi Y., Ohtori S. (2022). Determinants of fracture type in the proximal femur: Biomechanical study of fresh frozen cadavers and finite element models. Bone.

